# Spotlight on *hTERT* Complex Regulation in Cutaneous T-Cell Lymphomas

**DOI:** 10.3390/genes14020439

**Published:** 2023-02-08

**Authors:** Joana Ropio, Martina Prochazkova-Carlotti, Rui Batista, Ana Pestana, Alain Chebly, Jacky Ferrer, Yamina Idrissi, David Cappellen, Cecília Durães, Paula Boaventura, João Vinagre, Lamia Azzi-Martin, Sandrine Poglio, José Cabeçadas, Manuel António Campos, Marie Beylot-Barry, Manuel Sobrinho-Simões, Jean-Philippe Merlio, Paula Soares, Edith Chevret

**Affiliations:** 1 BRIC (BoRdeaux Institute of onCology), UMR1312, INSERM, University of Bordeaux, 33000 Bordeaux, France; 2 Institute of Biomedical Sciences of Abel Salazar, Porto University, 4050-313 Porto, Portugal; 3 Faculty of Veterinary Medicine, Lusófona University, 1749-024 Lisbon, Portugal; 4 Institute for Research and Innovation in Health (I3S), Porto University, 4200-135 Porto, Portugal; 5 Institute of Molecular Pathology and Immunology of the University of Porto (IPATIMUP), Cancer Biology Group, Porto University, 4200-465 Porto, Portugal; 6 Faculty of Medicine, Porto University, 4200-319 Porto, Portugal; 7 Medical Genetics Unit, Faculty of Medicine, Saint Joseph University, Beirut 1104 2020, Lebanon; 8 Higher Institute of Public Health, Saint Joseph University, Beirut 1104 2020, Lebanon; 9 Tumor Bank and Tumor Biology Laboratory, Bordeaux University Hospital, 33075 Bordeaux, France; 10 UFR des Sciences Médicales, Bordeaux University, 33076 Bordeaux, France; 11 Dermatology Departement, Instituto Português de Oncologia de Lisboa (IPO-L), 1099-023 Lisbon, Portugal; 12 Centro Hospitalar Vila Nova de Gaia/Espinho, E.P.E., Dermatology Departement, 4434-502 Vila Nova de Gaia, Portugal; 13 Dermatology Department, Bordeaux University Hospital, 33075 Bordeaux, France; 14 Department of Pathology, Faculty of Medicine, Porto University, 4200-319 Porto, Portugal

**Keywords:** CTCL, telomerase, hTERT, promoter mutations, SNP, splicing variants

## Abstract

As a major cancer hallmark, there is a sustained interest in understanding the telomerase contribution to carcinogenesis in order to therapeutically target this enzyme. This is particularly relevant in primary cutaneous T-cell lymphomas (CTCL), a malignancy showing telomerase dysregulation with few investigative data available. In CTCL, we examined the mechanisms involved in telomerase transcriptional activation and activity regulation. We analyzed 94 CTCL patients from a Franco-Portuguese cohort, as well as 8 cell lines, in comparison to 101 healthy controls. Our results showed that not only polymorphisms (SNPs) located at the promoter of human telomerase reverse transcriptase (*hTERT)* gene (rs2735940 and rs2853672) but also an SNP located within the coding region (rs2853676) could influence CTCL occurrence. Furthermore, our results sustained that the post-transcriptional regulation of *hTERT* contributes to CTCL lymphomagenesis. Indeed, CTCL cells present a different pattern of *hTERT* spliced transcripts distribution from the controls, mostly marked by an increase in the *hTERT* β+ variants proportion. This increase seems to be associated with CTCL development and progression. Through *hTERT* splicing transcriptome modulation with shRNAs, we observed that the decrease in the α-β+ transcript induced a decrease in the cell proliferation and tumorigenic capacities of T-MF cells in vitro. Taken together, our data highlight the major role of post-transcriptional mechanisms regulating telomerase non canonical functions in CTCL and suggest a new potential role for the α-β+ *hTERT* transcript variant.

## 1. Introduction

Telomere shortening is considered to be a control mechanism limiting cells’ replicative capacity, inducing senescence once a critical level of shortening has occurred [1]. Therefore, cancer cells’ continuous proliferation requires the activation of a telomere maintenance mechanism. About 85% of human cancer cells activate the telomerase enzyme that adds telomeric repeats to chromosome ends, while the remaining 15% activate the Alternative Lengthening of Telomeres (ALT) mechanism [2,3]. Although numerous factors are involved in telomerase activity, *hTERT* (human TElomerase Reverse Transcriptase, the enzymes’ catalytic subunit) expression is its limiting factor in many cancers [4]. Beside its activity on telomere elongation, *hTERT* is also implicated in tumor formation and progression, since its expression is determinant to cell immortalization and resistance to senescence and apoptosis [5,6,7]. These telomere-independent functions are considered as telomerase non-canonical functions [8]. Thus, the regulation of the *hTERT* complex in cancer cells is highly controlled, involving numerous steps such as transcriptional and post-transcriptional mechanisms [9,10,11].

**Figure 1:**
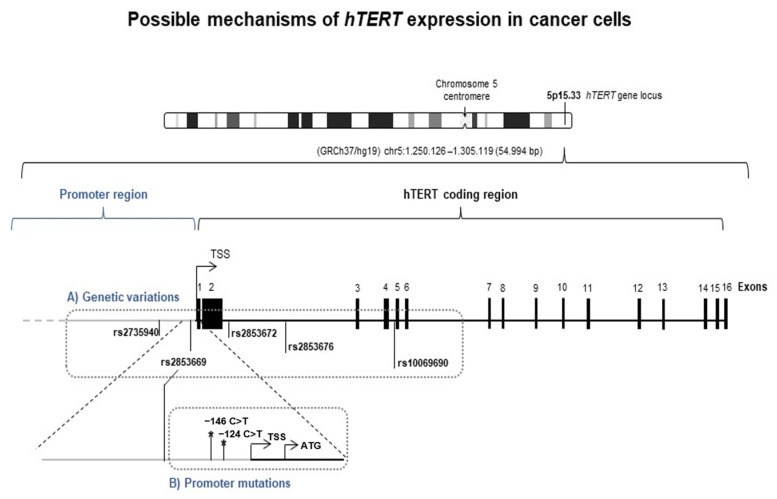
Possible mechanisms of *hTERT* expression in cancer cells. Telomerase activity in cancer cells is related to the acquired expression of *hTERT* gene located at the short arm of chromosome 5. *hTERT* transcription activation may be due to germline genetic variations and promoter hotspot mutations. (**A**) Localization of *hTERT* germline genetic polymorphisms associated with cancer risk in promoter (rs2735940 and rs2853669) and in gene coding region (rs2853672, rs2853676, rs10069690). The 16 exons of *hTERT* gene are represented by black vertical bars. (**B**) *hTERT* promoter hotspot mutations (*) −124 bp and −146 bp upstream the ATG. Genomic coordinates are based on build 37 (GRCh 37, hg19/Human). *hTERT:* human telomerase reverse transcriptase; bp: base pair.

Non-coding mutations within the *hTERT* core promoter provided the first mechanism of cancer-specific telomerase (re)activation (Figure 1B) [12,13]. Two hotspot mutations, located at −124 C > T and −146 C > T (from the ATG start site), generate a new consensus binding site for ETS/TCFs transcription factors, which increases the *hTERT* transcription and activity two to four times [14]. This mechanism has strong clinical implications conferring a worse prognosis and poor survival in many cancers [2,15,16].

**Figure 2:**
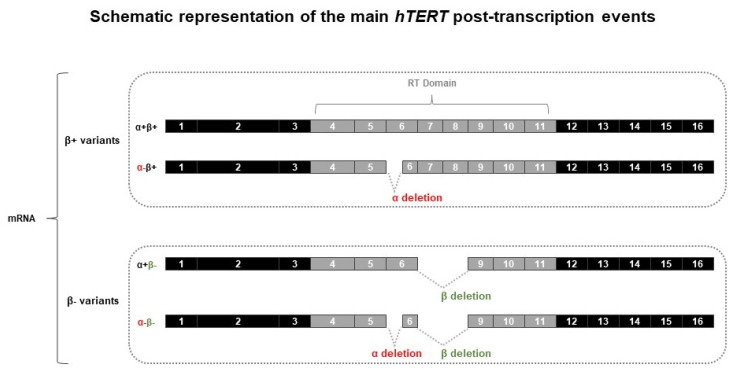
Schematic representation of the main *hTERT* post-transcriptional events. The two main alternative splicing sites in *hTERT* RT domain are the α splice site in exon 6, which produces a 36 bp in-frame deletion; and the β splice site in exons 7 and 8, which results in a 182 bp deletion. Four possible combinations of *hTERT* alternative splicing are possible: α+β+, α−β+ (mentioned as β+ variants), and α+β− and α−β− (mentioned as β− variants). *hTERT:* human telomerase reverse transcriptase; RT: reverse transcriptase.

At the post-transcriptional level, *hTERT* pre-mRNA is subject to alternative splicing, which generates proteome diversity with different biological functions. To date, around 20 transcript variants have been identified [11,17,18]. Cells exhibiting telomerase activity co-express, at significant levels, different *hTERT* transcripts and evidence exists that *hTERT* alternative splicing may play a critical role in the regulation of telomerase activity [19,20,21]. The two most studied *hTERT* alternative splicing events occur within the telomerase catalytic reverse transcriptase (RT) domain, at α and β sites (Figure 2). The α site originates from a 36-long base pair (bp) in-frame deletion on exon 6, while the β site encompasses a 183 bp long deletion from exons 7 and 8 that generates a truncated protein (Figure 2) [22]. Splicing at α and β sites can occur separately or in combination, generating either α+β+, α−β+, α+β− or α−β− *hTERT* transcripts (Figure 2). Only the full-length RT domain *hTERT* transcript, α+β+, exhibits telomerase activity [23], while the other variants may exhibit different functions. The α−β+ variant is a negative regulator of telomerase activity [22]. The α+β− protein, as it conserves the RNA-binding motif, can also act as a negative regulator of telomerase activity alongside its capacity to protect cancer cells from cell death by apoptosis [22,24,25]. While the α−β− *hTERT* variant is described as the less expressed transcript, no specific function has been assigned to this *hTERT* variant yet [26].

Genetic variants of *hTERT* were also found to play a crucial role in the risk and prognosis of human cancers. Indeed, based on genome-wide association studies (GWAS), single nucleotide polymorphisms (SNPs) within the *hTERT* locus (5p15.33) have been consistently associated with an increased risk for developing various types of cancers [2,27]. *hTERT* SNPs are located either within its promoter, or in intronic and also exonic regions (Figure 1A). Usually, *hTERT* SNPs do not generate deleterious coding alleles; however, they are reportedly associated with telomere lengthening, such as rs2853672, rs2853676, and rs10069690 polymorphisms [28,29,30,31,32,33]. Meanwhile, polymorphic changes in the *hTERT* promoter sequence (rs2853669 and rs2735940) were found to influence the telomerase expression [34,35,36].

Primary cutaneous T-cell lymphomas (CTCL) are a heterogeneous group of non-Hodgkin lymphomas presenting in the skin with no evidence of extracutaneous disease at the time of diagnosis [37]. The most common subtypes, comprising 75% of CTCL, include mycosis fungoides (MF) (representing around 50% of CTCL cases), Sézary syndrome (SS), and CD30+ lymphoproliferative disorders (LPDs) [37]. Patients with MF usually experience an indolent disease with a 5 year disease-specific survival (DSS) of 88% [38]. However, a minority undergo a process of large-cell transformation (transformed mycosis fungoides, T-MF), which often heralds a more aggressive disease, with the cancer spreading to lymph nodes and/or internal organs [39,40]. SS is a rare aggressive leukemic type of CTCL, traditionally defined by the triad of pruritic erythroderma, generalized lymphadenopathy, and clonally related neoplastic T cells with cerebriform nuclei (Sézary cells) in the skin, lymph nodes, and peripheral blood [37,41]. The 5-year DSS in SS is around 36% [42]. LPDs comprise a spectrum of conditions with similar histologic and molecular features, but different clinical presentations. They include lymphomatoid papulosis (LyP) and primary cutaneous anaplastic large cell lymphomas (c-ALCL), both with a favorable prognosis and a 5-year DSS greater than 95% [37,43].

The deregulation of the telomerase expression and telomere length is a common feature of hematological diseases, including CTCL [44,45,46]. We previously demonstrated that telomerase is expressed in different CTCL subtypes (c-ALCL, T-MF and SS) and showed that short telomeres are a hallmark of the aggressive subtypes (T-MF and SS) [46]. Furthermore, our team also showed that besides the maintenance of the telomere length, telomerase exerts additional functions in CTCL [46].

In this work, we aimed to deepen the knowledge on the *hTERT* regulation in CTCL cells. We investigated the *hTERT* expression-regulating mechanism in order to understand the regulation of the telomerase functions. Our results represent a step forward towards a better understanding of the telomerase reactivation in neoplastic CTCL cells, taking into account the potential future therapeutic implications against telomerase in cancer cells.

## 2. Methods

### 2.1. Patients and Healthy Controls

All tumors included in this study, obtained from French and Portuguese institutions, were classified according to the criteria of the World Health Organization–European Organization for Research and Treatment of Cancer (WHO-EORTC) [37].

Tumor DNA and peripheral blood samples from 61 patients were collected from the dermatology department at Bordeaux University Hospital Center (CHU) (France) as well as 33 representative formalin-fixed paraffin-embedded (FFPE) samples from the pathology archives at Instituto Português de Oncologia de Lisboa (IPO-L) and Centro Hospitalar Vila Nova de Gaia/Espinho (Portugal). A total of 94 tumor samples were analyzed (29 ≤ age ≤ 87, mean age 65), including 22 LPDs (14 cALCL and 8 LyP), 39 MF (24 classic MF and 15 T-MF), and 33 SS. The institutional review board approved the manipulation of the CTCL patients’ samples (DC-2015-412). Peripheral blood from 101 healthy donors (24 ≤ age ≤ 85, mean age 60) was obtained from the Etablissement Français du Sang, Bordeaux (DC 2015 2412-18PLER012), and CHU of Bordeaux (France). Peripheral blood mononuclear cells (PBMC) from patients and healthy donors were isolated by PANCOLL^®^ density gradient centrifugation (PAN-Biotech, Aidenbach, Germany).

### 2.2. CTCL Cell Lines

Experiments were performed on six classical CTCL cell lines, including four cALCL: Mac1, Mac2A, Mac2B (DSMZ), and FEPD (Prof. G. Delsol, Toulouse, France); one T-MF: MyLa (Dr K. Kaltoft, Aarhus, Denmark); and one SS: Hut78 (ATCC). Furthermore, we included two SS cell lines newly developed in our laboratory: L1 and L2 [47]. A human T-cell leukemia cell line, 1301 (Sigma-Aldrich), was used as a positive control for the amplification of the *hTERT* RT domain splicing variants. The cells were cultured in an RPMI 1640 media (Gibco, Waltham, MA, USA) supplemented with 1% penicillin–streptomycin (Gibco) and 10% fetal bovine serum (Eurobio, Les Ulis, France), except for the L1 and L2 cell lines, which were cultured as previously described [47]. All cell lines were incubated at 37 °C with 5% CO_2_ and regularly tested for mycoplasma contamination.

### 2.3. Telomerase Activity Estimation

The telomerase activity was assessed from the protein extracts by means of the TRAPeze^®^ RT telomerase detection kit (Sigma-Aldrich, Saint-Quentin-Fallavier, France), according to the manufacturer’s instructions with some modifications, as previously described [46].

### 2.4. Nucleic Acid Isolation

Genomic DNA was extracted by the salt precipitation method, previously detailed in [48]. The total RNA was isolated using the Direct-zol™ RNA MiniPrep kit (ZYMO Research, Breisgau, Germany). Both the DNA and RNA concentrations were measured by a NanoDrop 2000 spectrophotometer. The DNAs quality was analyzed by classic agarose gel electrophoresis and the RNA quality was analyzed on Agilent 2200 TapeStation system. The DNA was stored at −20 °C and the RNA was stored at −80 °C, until a further genetic analysis.

### 2.5. Telomere Length Measurement

The telomere length was calculated from the DNA samples by means of the Absolute Human Telomere Length Quantification qPCR Assay Kit (ScienCell, Carlsbad, CA, USA), as previously described [48].

### 2.6. hTERT Hotspot Promoter Mutations Detection

The −146:C > T and −124:C > T *hTERT* promoter mutations were screened by PCR followed by direct Sanger sequencing in DNA samples, as previously described [15]. Primers used are available in Supplementary Table S1.

### 2.7. hTERT SNPs Genotyping

The SNPs were genotyped either by an allele-specific PCR or by TaqMan probes. *hTERT* SNPs rs2735940, rs2853672, rs2853676, and rs10069690 were genotyped by an allele-specific PCR. The allele-specific reactions were analyzed in the DNA samples, with each individual forward and reverse primer sets (Supplementary Table S1) and Takyon^TM^ No Rox SYBR^®^ MasterMix dTTP Blue (Eurogentec, Seraing, Belgium), according to the manufacturer’s instructions. All SNP amplification reactions were carried out in a Stratagene Mx3005P system (Agilent Technologies, Santa Clara, CA, USA) and followed the same qPCR program setup: initial denaturation step at 95 °C for 3 min, followed by 40 cycles of 95 °C for 20 s and 60 °C for 20 s, with signal acquisition. *hTERT* rs2853669 polymorphism was analyzed by means of the TaqMan SNP genotyping assay (Life Technologies) in an ABI Prism 7500 Fast system (Life Technologies, Carlsbad, CA, USA).

### 2.8. hTERT Splicing Variants Expression

In total, 1 µg of the total RNA was reverse transcribed using the SuperScript II reverse transcriptase kit (Invitrogen, Waltham, MA, USA), following the manufacturer’s instructions. The complementary DNA (cDNA) was amplified using specific primers (Supplementary Table S1) and Takyon^TM^ No Rox SYBR^®^ MasterMix dTTP Blue (Eurogentec, Seraing, Belgium). Amplifications were carried out on a Stratagene Mx3005P system and analyzed with MxPro 4.01 qPCR software Stratagene (Agilent Technologies, Santa Clara, CA, USA). The expression quantification was normalized to the expression level of the TATA box-binding protein (*TBP*) reference gene. The *hTERT* splicing variants were amplified as follows: initial denaturation step at 95 °C for 3 min, followed by 45 cycles of 95 °C for 20 sec and 60 °C for 60 s with signal acquisition. The 1301 cell line was used as a positive control and its dissociation curves were used as the reference (Supplementary Figure S1).

### 2.9. Lentiviral shRNA Construction and Production

Constructs sh1, sh2 (targeting *hTERT* β site), and the sh control (non-targeting) were cloned into a *pLKO.1-Tomato* (Addgene, Watertown, MA, USA) vector at the AgeI/*Eco*RI sites. shRNA primer sequences are available in Supplementary Table S2. Lentiviral vector construction maps are available in Supplementary Figure S2. Lentiviral vectors were used to transfect the HEK293T cells at Bordeaux University Vectorology platform, to induce viral production.

### 2.10. Lentiviral Cell Transduction

MyLa cells were transduced with the appropriate volume of virus to obtain 33% of transduced cells. After 10 days, positively transduced cells were selected by flow cytometry on the BD FACSAria^TM^ III sorter (BD Biosciences, San Jose, CA, USA).

### 2.11. Cell Proliferation Analysis

The cell proliferation capacity of transduced MyLa cells was measured by direct cell-counting in a hemocytometer (KOVA, Garden Grove, CA, USA). Furthermore, 2 × 10^5^ cells per well were seeded into 12-well plates and counted after 2, 5, 8, and 12 days of culture.

### 2.12. Cell Clonogenicity Analysis

Transduced MyLa cells were placed in soft agar in 6-well plates and tested for their capacities to form cell colonies. The soft agar technique was previously described [46].

### 2.13. Statistical Analysis

Statistical analyses were performed on GraphPad Prism software (version 8.0.1 (244)) and on SPSS 23 (IBM SPSS Statistics). *hTERT* RT domain splicing variants statistical analyses were performed on GraphPad Prism software. Data from the patients were collected from triplicate reactions from each sample. Data from the cell lines were collected from triplicate reactions from two independent biological experiments. The results were presented as the mean ± standard deviation. A paired Mann–Whitney test (nonparametric *T*-test) was used to compare the results.

Genotype frequencies for *hTERT* SNPs were obtained using SPSS 23. The compliance of the alleles with the Hardy–Weinberg equilibrium was measured at the level of the control population using a χ2 test. The comparison of the genotype frequencies between the groups was assessed by the unconditional logistic regression with SPSS 23. The odds ratios (OR) with respective confidence intervals (95% CI) were calculated considering the genotypic and the dominant models of inheritance. The significance level of the *p*-value was set to <0.05.

## 3. Results

### 3.1. hTERT SNPs Are Associated with CTCL Risk in Patient Cells

Two *hTERT* promoter SNPs, rs2735940 T > C and rs2853669 T > C, along with three *hTERT* intronic SNPs, rs2853672 G > T, rs2853676 G > A, and rs10069690 C > T (Figure 1A), were genotyped in 101 healthy controls, as well as in 66 CTCL patients and 8 cell lines (Table 1). The distribution of all SNP genotypes in the control group were in accordance with Hardy–Weinberg equilibrium: rs2735940 T > C (χ^2^ = 0.06, *p* > 0.05), rs2853669 T > C (χ^2^ = 0.3, *p* > 0.05), rs2853672 G > T (χ^2^ = 0.3, *p* > 0.05), rs2853676 G > A (χ^2^ = 0.3, *p* > 0.05), and rs10069690 C > T (χ^2^ = 1.73, *p* > 0.05) (Table 1).

The results showed that the rs2853669 SNP genotype distribution was not different between the patients and the controls (Table 1). The genotyping of rs10069690 SNP revealed a decrease in the prevalence of the TT genotype in CTCL patients (OR (95%CI) = 0.14 (0.017–1.21), *p* = 0.074), although this was not statically significant. Hence, no impact on the CTCL risk was observed for these two *hTERT* SNPs. On the other hand, the genotyping of rs2735940 SNP revealed that this SNP can impact the risk of CTCL. Indeed, rs2735940 TC and CC genotypes were significantly more prevalent in the patients than in the controls (OR (95%CI) = 3.00, *p* = 0.010 and OR (95%CI) = 3.79, *p* = 0.011, respectively). Hence, the CTCL risk was significantly increased in the rs2735940 C allele carriers (OR (95%CI) = 3.20, *p* = 0.004) (Table 1). The CTCL risk was also significantly influenced by rs2853672 and rs2853676 SNPs when using the dominant model (Table 1). Additionally, for the rs2853672, the T allele enhanced the risk for CTCL by two times (OR (95%CI) = 2.18, *p* = 0.039); and for the rs2853676, the minor allele A was associated with a lower risk for CTCL (OR (95%CI) = 0.46, *p* = 0.028) (Table 1).

It is worth mentioning that each CTCL cell line presented a specific combination of these five polymorphisms (Supplementary Table S3).

### 3.2. hTERT Transcription Regulation in CTCL: hTERT Promoter Mutations Are a Rare Event in CTCL Patients

As the mechanism underlying the telomerase activation in CTCL started to be uncovered [49,50], we investigated the occurrence of the non-coding mutations within the *hTERT* core promoter.

The occurrence of the two hotspot *hTERT* promoter mutations, −124C > T and −146C > T (from the ATG) (Figure 1B), were first studied in the CTCL cell lines. Out of the eight cell lines screened, only one, the T-MF-derived cell line MyLa, harbored the −146C > T mutation (Table 2). The mutation was homozygous. This encouraged us to retrospectively investigate these mutations in a cohort of 8 patients with a history of MF that transformed to T-MF, along with 18 LPDs, 24 MF with no progression, and 17 SS patients. Among the CTCL cohort of 67 patients, only one SS patient harbored the −146 C > T mutation, which represented 5.9% of SS cases and 1.5% of all CTCL patients (Table 2).

### 3.3. hTERT Post-Transcription Regulation in CTCL Cells

We previously demonstrated that besides the maintenance of the telomere length, telomerase exerts additional functions in CTCL [46]. Therefore, we investigated the *hTERT* post-transcriptional regulation through an alternative splicing mechanism in order to investigate the implication of such mechanisms in the telomerase regulation in CTCL cells.

#### 3.3.1. hTERT Is Subjected to Alternative Splicing in CTCL Cells

**Figure 3:**
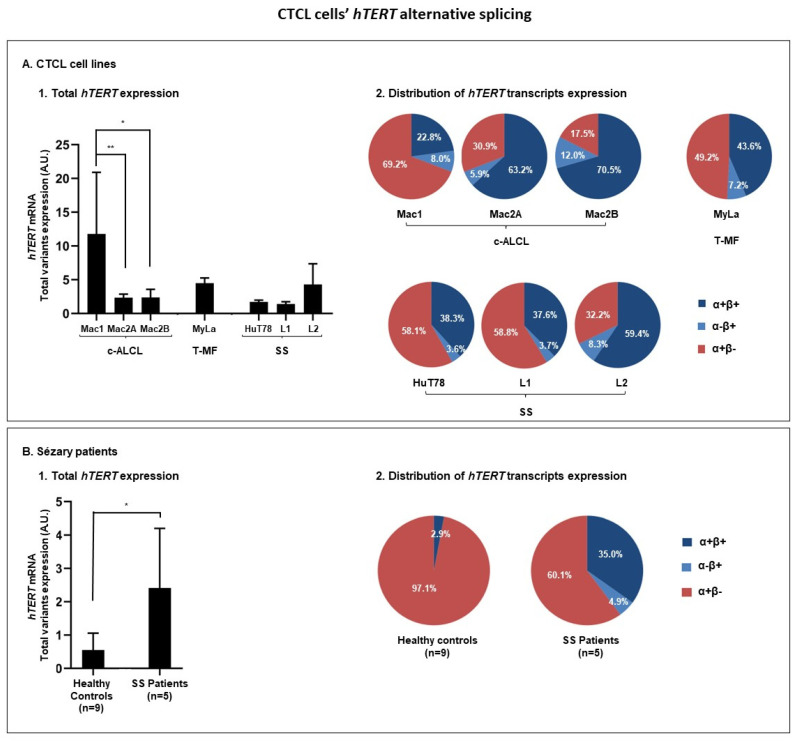
CTCL cells’ *hTERT* alternative splicing. (**A**) *hTERT* RT domain transcriptome analyzed in CTCL cell lines representative of different CTCL subtypes: Mac1, Mac2A, and Mac2B for c-ALCL, MyLa for T-MF, and HuT78, L1 and L2 for SS. (**A1**) *hTERT* transcripts expression levels. (**A2**) Distribution of *hTERT* transcripts expression. β+ variants are presented in blue and β− variants in red. (**B**) *hTERT* RT domain transcriptome analyzed in peripheral blood mononuclear cells from five SS patients, in comparison with nine healthy controls. (**B1**) *hTERT* transcripts expression. (**B2**) Distribution of *hTERT* transcripts expression. β+ variants are presented in blue and β- variants in red. A.U.: arbitrary unit; CTCL: cutaneous T-Cell lymphomas; c-ALCL: primary cutaneous anaplastic large cell lymphomas; T-MF: transformed mycosis fungoides; SS: Sézary syndrome; *hTERT*: human telomerase reverse transcriptase; RT: reverse transcriptase. Statistical significance: * *p* < 0.05 and ** *p* < 0.01.

First, we studied the cell lines representative of the most common subtypes of CTCL: Mac1, Mac2A, and Mac2B for c-ALCL, MyLa for T-MF, and for SS HuT78, L1 and L2 (Figure 3A). Mac1, the only cell line with indolent behavior, was the cell line that expressed the highest levels of total *hTERT* variants (sum of α+β+, α−β+ and α+β−) (Figure 3A1).

The Mac2A and Mac2B cell lines were established from the same patient as Mac1, but during the aggressive terminal phase of the disease, and they express less total *hTERT* variants than Mac1 (Figure 3A1 and Table 3). Concordantly, Mac2A and Mac2B exhibited an *hTERT* transcripts distribution significantly different from Mac1 (Figure 3A2). Indeed, Mac2A and Mac2B presented a significant decrease in the α+β− variant, along with an important increase in the α+β+ variant, compared to Mac1 (Figure 3A2 and Table 3). MyLa, HuT78, L1, and L2, four aggressive CTCL cell lines, all expressed lower levels of total *hTERT* variants compared to the cell line with indolent behavior, Mac1 (Figure 3A1). Additionally, while comparing with Mac1 cells, the T-MF and SS cell lines presented a reduced α+β− variant expression, and an increased α+β+ variant expression. Interestingly, we did not detect any α−β− *hTERT* variant expression in all CTCL cell lines analyzed (Figure 3A2).

Then, we studied the *hTERT* splicing transcriptome in five SS patients compared to nine healthy donors used as the controls (Figure 3B). SS patients expressed significantly more total *hTERT* variants than the controls (Figure 3B1 and Table 3). Regarding the *hTERT* transcripts distribution, the healthy controls presented an almost exclusive expression of the *hTERT* α+β− variant (Figure 3B2 and Table 3). In contrast, SS patients presented a significative increase in the *hTERT* β+ variants proportion (α+β+ and α−β+) *(p* = 0.0126), mainly α+β+ (Figure 3B2 and Table 3). Indeed, compared to the healthy controls, the expression of the α+β− *hTERT* variant was significatively reduced in SS patients (*p* = 0.0290), while the expression of the α+β+ variant was increased (*p* = 0.2507) (Figure 3B2 and Table 3). Interestingly, the expression of the α−β+ variant was restricted to tumor cells (*p* = 0.0050) (Figure 3B2 and Table 3). The α−β− *hTERT* variant was neither detected in healthy controls nor in SS patient cells.

#### 3.3.2. Modulation of RT Domain Transcriptome

**Figure 4:**
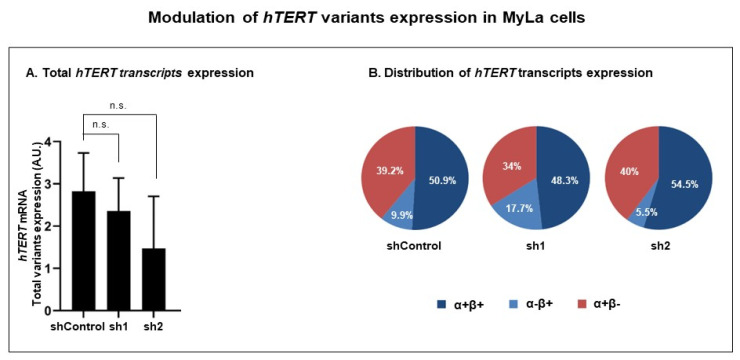
Modulation of *hTERT* variants expression in MyLa cells. MyLa cells were transduced with shRNAs (sh1 and sh2) targeting *hTERT* β variants, and with a non-targeting shRNA (sh control). (**A**) *hTERT* transcripts expression. (**B**) Distribution of *hTERT* transcripts expression. β+ variants are presented in blue and β− variants in red. A.U.: arbitrary unit; shRNAs: short hairpin RNAs; hTERT: human telomerase reverse transcriptase.

As we found *hTERT* variants differently expressed in CTCL cells with indolent and aggressive behavior, we modulated the *hTERT* RT domain transcriptome in an aggressive cell line, with shRNAs targeting the *hTERT* β variants (Figure 4) in order to modulate the expression of the *hTERT* variants. We selected the MyLa cell line that consistently expresses both *hTERT* mRNA [46] and total *hTERT* variants (Figure 3A).

Although neither shRNA1 (sh1) nor shRNA2 (sh2) impacted significantly the total level of the *hTERT* variants (Figure 4A and Table 4), they induced a modulation of the *hTERT* transcripts distribution (Figure 4B and Table 4). The ShRNAs (sh1 and sh2) used neither impacted the expression of the α+β− transcript nor the expression of the full transcript, α+β+ (Figure 4B and Table 4). On the other hand, sh1 induced a significant increase in the expression of the α−β+ transcript (*p* = 0.0237), while sh2 induced a significant decrease in this transcript’s expression (*p* = 0.0003) (Figure 4B and Table 4).

#### 3.3.3. Modulation of hTERT Transcriptome Affects Telomerase Non-Canonical Functions

**Figure 5:**
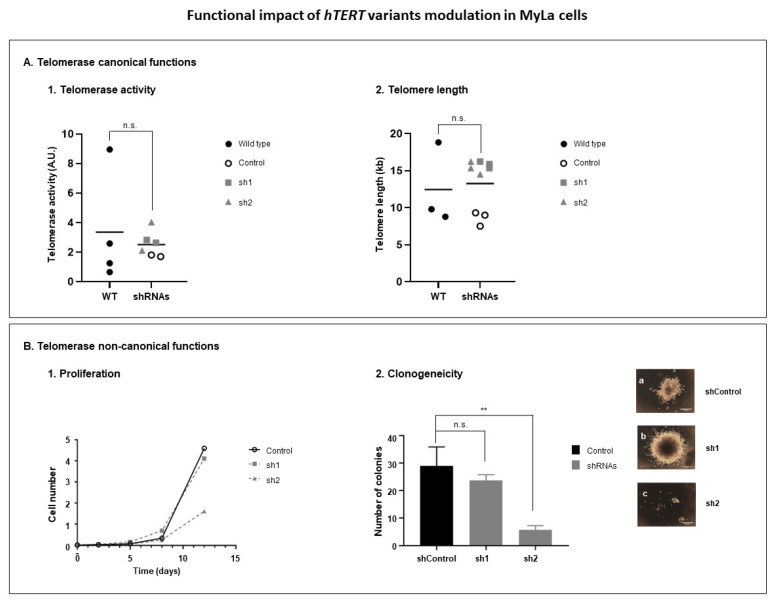
Functional impact of *hTERT* variants modulation in MyLa cells. After one month of transduction with shRNAs, in MyLa cells, (**A**) telomerase canonical functions were evaluated through the assessment of (**A1**) telomerase activity and (**A2**) telomere length. In the same transduced cells, (**B**) telomerase non-canonical functions were evaluated, through the assessment of (**B1**) cell proliferation and (**B2**) cell colony formation capacities. Figures (**a**–**c**), show colony images of MyLa control cells as well as transduced MyLa cells with sh1 and sh2, respectively. A.U.: arbitrary unit; shRNAs: short hairpin RNAs; *hTERT*: human telomerase reverse transcriptase. **: *p* < 0.05.

The functional impact of the *hTERT* transcriptome was evaluated on telomerase activities (Figure 5). The impact was first evaluated on the telomerase canonical functions, related with the telomere length. Thus, the telomerase activity and telomere length were evaluated in MyLa transduced cells compared with non-transduced cells (Wild type, WT) (Figure 5A). Concerning the telomerase activity, no impact was observed (*p* = 0.6095) (Figure 5A1). Concordantly, no impact was observed on the telomere length (*p* = 0.9727) (Figure 5A2). We then analyzed the impact of the *hTERT* transcriptome modulation on the telomerase non-canonical functions, focusing on the cell proliferation and cell clonogenic capacities (Figure 5B). On MyLa cells, sh1 induced no effect on the cell proliferative capacities, while on the cells transduced with sh2, we observed a strong decrease in the cell proliferative capacities (Figure 5B1). Concordantly, sh1 induced no effect on the cell clonogenic capacities (*p* = 0.2707), while sh2 significantly decreased the number of colonies formed by MyLa cells (*p* = 0.0047) (Figure 5B2).

## 4. Discussion

As a major hallmark in cancer, telomerase has been extensively investigated in order to understand its contribution in cancer, with the hope that it could potentially be targeted for cancer treatment [51,52]. This is particularly relevant in CTCL, a group of malignancies known to be telomerase-positive [46]. However, little is known about the molecular basis of the *hTERT* transcriptional activation and regulation in CTCL, which can be potentially useful for diagnostic, prognostic, and therapeutic purposes.

Genome-wide association studies (GWAS) of cancer etiology have identified variants in the *hTERT* gene that can impact the outcome of numerous subtypes of cancers. Such information paved the way to understand the associations between common genetic variants and human diseases or specific phenotypes [27,53]. Based on our results, CTCL can be added to the list of cancers to which *hTERT* single nucleotide polymorphisms (SNPs) are associated with disease risk. *hTERT* SNPs do not generate deleterious coding alleles; instead, they are reportedly associated with the telomere length [28,29,30,31,32,33]. Advanced stage CTCL present telomere length deregulation, which is a coherent argument to find *hTERT* SNPs associated with disease risk [46]. Additionally, *hTERT* promoter SNPs can influence the gene expression by altering the promoter transcription activity [27,53]. Thus, our data are in favor of a potential role for *hTERT* SNP rs2735940 T>C in influencing the *hTERT* expression. More studies are needed in order to establish this correlation and investigate the role of rs2735940 T>C in CTCL clinical development.

Previous investigation revealed that copy number alteration/rearrangements of the *hTERT* locus are not involved in the telomerase activation in CTCL [46]. On the other hand, the occurrence of somatic mutations within the *hTERT* promoter, a recurring event described in several cancers, has never been investigated in CTCL cells [54]. Our results revealed that these hotspot mutations are rare in CTCL. Surprisingly, when detected, *hTERT* promoter mutation occurred at only one position (−146 bp from the ATG start site), and it was restricted to aggressive CTCL subtypes. Knowing that CTCL tumors are classified among hematologic malignancies, our results are in concordance with what is already published regarding the statement that somatic *hTERT* promoter mutations are rare in hematologic cancers [15,55,56].

Aside the telomerase canonical function on the telomere elongation, telomerase also exhibits other non-canonical functions largely implicated in the initiation and progression of cancer [46,57]. The pre-mRNA alternative splicing of *hTERT* is one of the mechanisms that, despite regulating the telomerase activity, may also play a role in other cellular functions [11,18]. Herein, we reported, for the first time, that *hTERT* is subjected to alternative splicing in CTCL cells. Focusing the analysis of the *hTERT* expression on the expression of *hTERT* α and β variants, we could discriminate between indolent and aggressive CTCL subtypes, and we could also observe that the distribution of *hTERT* transcripts in Sézary patients is substantially different from the healthy controls. It is worth mentioning the great diversity in the distribution of the *hTERT* transcripts in Sézary patients (Supplementary Figure S3), corroborating that the genetics of Sézary syndrome are diverse and complex [58,59,60]. Nevertheless, on the one hand, we observed an increase in the proportion of *hTERT* β+ variants (α+β+ and α−β+) in CTCL cells compared to the healthy controls. On the other hand, advanced tumor-stage CTCL cells presented a significant decrease in the α+β− expression, the most abundant *hTERT* transcript in healthy T lymphocytes and indolent tumor cells. Interestingly, the α−β+ transcript was exclusively observed in CTCL cells. Thus, we hypothesize that the increase in the *hTERT* β+ variants (α+β+ and α−β+) may be associated with disease development and progression. We believe that the sh1 construction did not produce reliable results; consequently, we focused on the results produced by the sh2 construction. Therefore, based on the functional impact of the *hTERT* transcriptome modulation that we observed with sh2, the α−β+ *hTERT* variant in particular, we suggest that α−β+ might play an important role in CTCL cells. Indeed, when we achieved to decrease its expression, we observed a significant decrease in the cell proliferation and clonogenic capacities. The need for tissue and tumor-specific TERT isoform investigations was clearly stated [61], and our work reinforced this statement and confirmed that further investigations are needed to corroborate and strengthen our findings.

Recently, it was demonstrated that regulatory T-cells may suppress the proliferation of target human and murine T- and B-lymphocytes, and also NK cells, in a contact-independent manner, involving the activation of TERT alternative splicing [11]. Additionally, there are several pharmacological drugs that can modulate *hTERT* alternative splicing, and they are being evaluated as anti-cancer therapies [62]. The small-molecule ligand 12459 and compound CX-5461 were reported to downregulate the telomerase activity by altering the *hTERT* splicing patterns in lung carcinoma and glioblastoma cell lines, respectively [63,64]. The currently available therapies in CTCL are reported to control the disease; meanwhile, the only curative option is stem cell transplantation [65]. According to the findings reported here, CTCL may be considered as good candidates to test, in vitro as a first step, the efficacy of *hTERT* alternative splicing modulating drugs. This study provides an insight regarding *hTERT* contribution in CTCL lymphomagenesis. It identifies a possible genetic predisposition to CTCL based on the *hTERT* genetic variants and excludes the *hTERT* promoter mutations as a relevant mechanism in the telomerase reactivation in CTCL. Furthermore, this study provides the first insight into the *hTERT* transcript pattern in different CTCL subtypes and provides a glance at the impact of the α−β+ *hTERT* variant on cancer cells’ proliferation and tumorigenic capacities, in vitro. Altogether, our results enhance our knowledge regarding the telomerase implication in CTCL and provide new insights into its potential therapeutic targeting in this pathology.

## Figures and Tables

**Table 1:** Genotypic frequencies of rs10069690 C > T, rs2853676 G > A, rs2853672 G > T, rs2853669 T > C and rs2735940 T > C *hTERT* polymorphisms in CTCL cell lines, CTCL patients, and healthy controls.

Locus/Genotype	Cell Lines *n* (%)	Controls *n* (%)	Patients *n* (%)	OR (95% CI)	*p*-Value
rs2735940	*n* = 8	*n* = 101	*n* = 66		
TT	2 (25)	40 (39.6)	11 (16.7)	1.00 ^a^	
TC	6 (75)	48 (47.5)	39 (59.1)	3.00 (1.31–6.89)	**0.010**
CC	0	13 (12.9)	16 (24.2)	3.79 (1.35–10.6)	**0.011**
Dominant model (C carrier vs. TT ^b^)		61 (60.4)/11 (39.6)	55 (83.3)/11 (16.7)	3.20 (1.44–7.08)	**0.004**
rs2853669	*n* = 8	*n* = 96	*n* = 66		
TT	0	40 (41.7)	28 (42.4)	1.00 ^a^	
TC	8 (100)	42 (43.7)	29 (43.9)	0.84 (0.41–1.75)	0.649
CC	0	14 (14.6)	9 (13.6)	0.80 (0.29–2.21)	0.660
Dominant model (C carrier vs. TT ^b^)		56 (58.3)/40 (41.7)	38 (57.6)/28 (42.4)	0.83 (0.42–1.64)	0.595
rs2853672	*n* = 8	*n* = 101	*n* = 66		
GG	2 (25)	38 (37.6)	16 (24.2)	1.00 ^a^	
GT	6 (75)	50 (49.5)	38 (57.6)	2.04 (0.94–4.44)	0.069
TT	0	13 (12.9)	12 (18.2)	2.67 (0.94–7.62)	0.063
Dominant model (T carrier vs. GG ^b^)		63 (62.4)/38 (37.6)	50 (75.8)/16 (24.2)	2.18 (1.04–4.58)	**0.039**
rs2853676	*n* = 8	*n* = 101	*n* = 66		
GG	4 (50)	47 (46.5)	40 (40.6)	1.00 ^a^	
AG	3 (37.5)	42 (41.6)	26 (39.4)	0.59 (0.29–1.19)	0.141
AA	1 (12.5)	12 (11.9)	0	0.00	0.999
Dominant model (A carrier vs. GG ^b^)		54 (53.5)/47 (46.5)	26 (39.4)/40 (60.6)	0.46 (0.23–0.92)	**0.028**
rs10069690	*n* = 8	*n* = 101	*n* = 66		
CC	0	50 (49.5)	36 (54.5)	1.00 ^a^	
CT	8 (100)	38 (37.6)	29 (43.9)	1.28 (0.64–2.57)	0.479
TT	0	13 (12.9)	1 (1.5)	0.14 (0.017–1.21)	0.074
Dominant model (T carrier vs. CC ^b^)		51 (50.5)/50 (49.5)	30 (45.5)/36 (54.5)	1.02 (0.52–1.98)	0.963

^a^ Reference value ^b^ reference genotype; *p*-values marked with bold indicate statistically significant *p*-values.

**Table 2:** *hTERT* promoter mutations analysis in CTCL cells.

	Mutation Rate (%)	Mutation
**Patients**	1/67 (1.5%)	
LPDs	0/18 (0%)	-
cALCL	0/10 (0%)	-
LyP	0/8 (0%)	-
MF	0/32 (0%)	-
MF	0/24 (0%)	-
T-MF	0/8 (0%)	-
SS	1/17 (5.9%)	−146 C > T
**Cell lines**	1/8 (12.5%)	
cALCL	0/4 (0%)	-
T-MF	1/1 (100%)	−146 C > T
SS	0/3 (0%)	-

CTCL: cutaneous T-cell lymphoma; LPDs: CD30+ lymphoproliferative disorders; cALCL: cutaneous anaplastic large cell lymphomas; LyP: lymphomatoid papulosis; MF: mycosis fungoides; T-MF: transformed mycosis fungoides; SS: Sézary syndrome; bp: base pair.

**Table 3:** *hTERT* transcripts expression level comparing indolent and aggressive phases in Mac cell lines, and also SS patients to healthy controls.

Cells	Total hTERT Transcripts (Sum of α+β+, α−β+, and α+β−)	α+β+	α−β+	α+β−
	% (*p*-Value)	% (*p* Value)		
Mac1	100	22.8	8.0	69.2
Mac2A	100 **(0.0022)**	63.2 (0.5887)	5.9 **(0.0022)**	30.9 **(0.0022)**
Mac2B	100 **(0.0260)**	70.5 (0.1667)	12.0 (0.0628)	17.5 **(0.0022)**
Healthy controls	100	2.9	0.0	97.1
SS patients	100 **(0.0120)**	35.0 (0.2507)	4.9 **(0.0050)**	60.1 **(0.0290)**

Statistical analysis of *hTERT* total transcripts expression and α+β+, α−β+, and α+β− hTERT transcripts proportions of Mac2A and Mac2B compared with Mac1 cells, as well as SS patients compared with healthy controls. *p*-values marked with bold indicate statistically significant *p*-values.

**Table 4:** *hTERT* transcripts expression analysis in transduced MyLa cells.

Cells	Total hTERT Transcripts (Sum of α+β+, α−β+ and α+β−)	α+β+	α−β+	α+β−
	% (*p* Value)	% (*p* Value)		
shControl		50.9	9.9	39.2
sh1	100 (0.1797)	48.3 (0.4857)	17.7 **(0.0237)**	34 (0.2196)
sh2	100 (0.1797)	54.5 (0.9448)	5.5 **(0.0003)**	40 (0.7255)

Statistical analysis of hTERT total transcripts expression and α+β+, α−β+, and α+β− hTERT transcripts proportions of MyLa cells transduced with sh1 and sh2 compared with MyLa shControl. P-values marked with bold indicate statistically significant p-values.

## Supplementary Materials

The following supporting information can be downloaded at: https://www.mdpi.com/article/10.3390/genes14020439/s1: Table S1. Primer sequences (5′ to 3′) and annealing temperature used for *hTERT* gene investigations: promoter mutations screening, SNPs genotyping, mRNA splicing variants expression. Table S2. Primer sequences (5′ to 3′) used for lentiviral short hairpin (sh) RNA vector cloning. Table S3. Genotyping results of rs2735940 T>C, rs2853669 T>C, rs2853672 G>T, rs2853676 G>A and rs10069690 C>T *hTERT* polymorphisms in CTCL cell lines. Figure S1. *hTERT* alternative splicing of 1301 cell line. Figure S2. Maps of pLKO.1 vectors used to insert *hTERT* β shRNA inserts. Figure S3. Sézary patient’s distribution of hTERT transcripts expression.
