# Supplementary material for: Spotlight on hTERT Complex Regulation in Cutaneous T-Cell Lymphomas
Source: Genes (Basel). 2023 Feb 8;14(2):439. doi: 10.3390/genes14020439 (PMC9956048; doi:10.3390/genes14020439)
Supplement: Supplementary file 1 [file genes-14-00439-s001.zip › genes-2142562-supplementary.pdf]

genes

MDPI

Supplemental data

Supplemental Tables

Supplemental Table S1. Primer sequences (5′ to 3′) and annealing temperature used for *hTERT* gene investigations: promoter mutations screening, SNPs genotyping, mRNA splicing variants expression.

Primers	Primer sequence	Annealing (°C)
***hTERT* promoter mutations**		
Fw pmutTERT	5′-CAGCGCTGCCTGAAACTC-3′	61.5
Rv pmutTERT	5′-GTCCTGCCCCTTCACCTT-3′	
hTERT* SNPs		
Fw (T) rs2735940	5′-GGATTTCTAGAAGAGCGGCC**T**-3′	
Fw (C) rs2735940	5′-GGATTTCTAGAAGAGCGGCC**C**-3′	60
Rv rs2735940	5′-TATGGAGCTAGCATTTGAACAG-3′	
Fw rs2853672	5′-AGGGTGCCTGCAGGTTACCTA-3′	
Rv (G) rs2853672	5′-CATATTGGCTGACCACGTACA**C**-3′	60
Rv (T) rs2853672	5′-CATATTGGCTGACCACGTACA**A**-3′	
Fw rs2853676	5′-TCGCCCCCTCACATGGATTG-3′	
Rv (G) rs2853676	5′-GAGGGAAGTCTGACGAATGC**C**-3′	60
Rv (A) rs2853676	5′-GAGGGAAGTCTGACGAATGC**T**-3′	
Fw rs10069690	5′-ACGGCTCCTGCACCCCAC-3′	
Rv (C) rs10069690	5′-ACACGGGATCCTCATGACA**C**-3′	60
Rv (T) rs10069690	5′-ACACGGGATCCTCATGACA**T**-3′	
**hTERT splicing variants** (Figure 2)		
Fw hTERT α+	5′-TGTACTTTGTCAAGGTGGATGTG-3′	
Fw hTERT α-	5′-CTGAGCTGTACTTTGTCAAGGAC-3′	
Fw hTERTβ	5′-CCAGCATCATCAAACCCCAG-3′	60
Rv hTERT β+	5′-GTACGGCTGGAGGTCTGTCAA-3′	
Rv hTERT β-	5′-GGCACTGGACGTAGGACGTGG-3′	
**Normalizing gene**		
Fw TBP	5′-CACGAACCACGGCACTGATT-3′	60
Rv TBP	5′-TTTTCTTGCTGCCAGTCTGGA-3′	

***In bold**, the specific SNP mismatch. Underlined, the common sequence mismatch added to each allele specific primer.

Supplemental Table S2. Primer sequences (5′ to 3′) used for lentiviral short hairpin (sh) RNA vector cloning.

Primers	Primer sequence
**sh *hTERT* 1**	
Fw **sh 1**	5′-CCGGGCATCAGGGGCAAGTCCTACGCTCGAGCGTAGGACTTGCCCCTGATGCTTTTTG-3′
Rv **sh 1**	5′-AATTCAAAAAGCATCAGGGGCAAGTCCTACGCTCGAGCGTAGGACTTGCCCCTGATGC-3′

**sh *hTERT* 2**	
Fw **sh 2**	5′-CCGGCAAGAGCCACGTCCTACGTCCCTCGAGGGACGTAGGACGTGGCTCTTGTTTTTG-3′
Rv **sh 2**	5′-AATTCAAAAACAAGAGCCACGTCCTACGTCCCTCGAGGGACGTAGGACGTGGCTCTTG-3′
**sh control**	
Fw **sh control**	5′-CCGGCAACAAGATGAAGAGCACCAACTCGAGTTGGTGCTCTTCATCTTGTTGTTTTT-3′
Rv **sh control**	5′-AATTAAAAACAACAAGATGAAGAGCACCAACTCGAGTTGGTGCTCTTCATCTTGTTG-3′

Supplemental Table S3. Genotyping results of rs2735940 T>C, rs2853669 T>C, rs2853672 G>T, rs2853676 G>A and rs10069690 C>T *hTERT* polymorphisms in CTCL cell lines.

		**Locus/genotype**				
**Pathology**	**Cell lines**	**rs2735940**	**rs2853669**	**rs2853672**	**rs2853676**	**rs10069690**
**c-ALCL**	**FEPD**	TT	TC	GG	GG	CT
	**Mac1**	TC	TC	GT	AG	CT
	**Mac2A**	TC	TC	GT	AG	CT
	**Mac2B**	TC	TC	GT	AG	CT
**T-MF**	**MyLa**	TT	TC	GG	AA	CT
**SS**	**HuT78**	TC	TC	GT	GG	CT
	**L1**	TC	TC	GT	GG	CT
	**L2**	TC	TC	GT	GG	CT

cALCL: cutaneous anaplastic large cell lymphomas. T-MF: transformed mycosis fungoides. SS: Sézary syndrome

Supplemental Figure

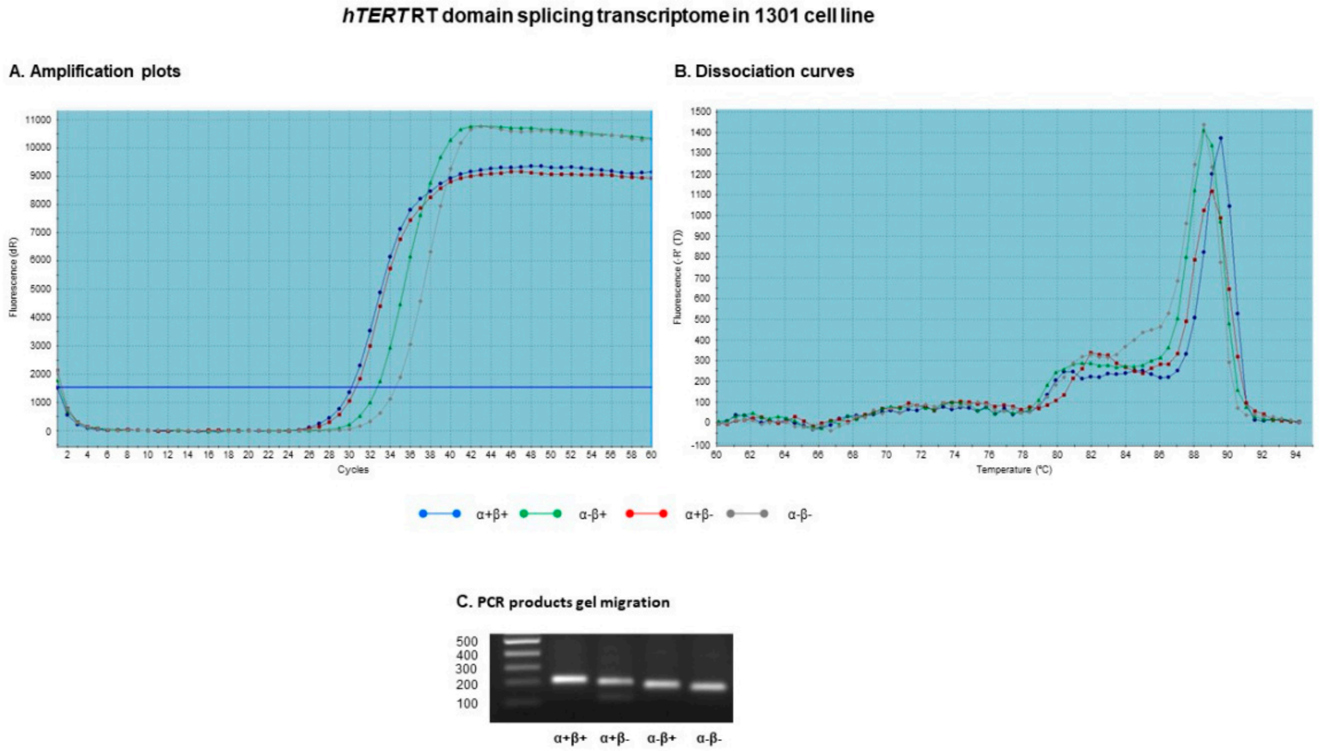

Supplemental Figure S1. *hTERT* alternative splicing of 1301 cell line. 1301 cell line was used as a positive control for amplification by RTqPCR of *hTERT* alternative spliced transcripts. A) Amplification plots of α+β+ (blue), α-β+ (green), α+β- (red) and α-β- (gray) transcripts, and B) corresponding dissociation curves. C) PCR products were run in a 2% agarose gel to confirm the attended weights of amplified products α+β+ (202 pb), α-β+ (189 pb), α+β- (172 pb) and α-β- (159 pb).

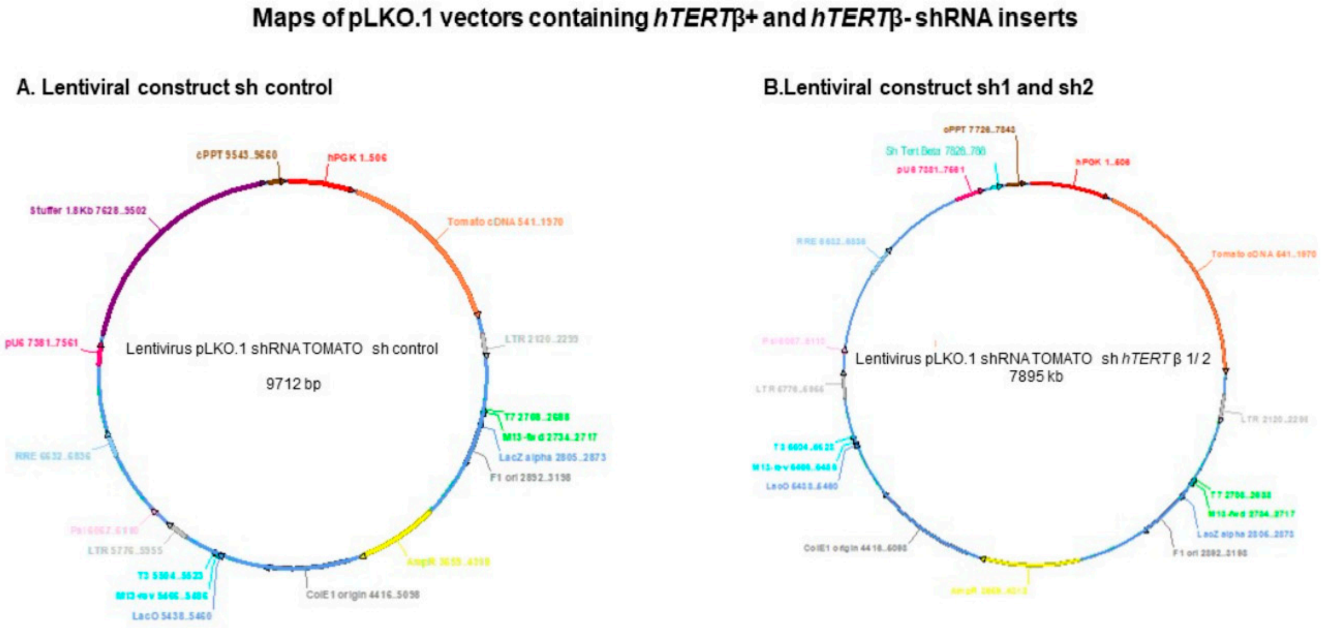

Supplemental Figure S2. Maps of pLKO.1 vectors used to insert *hTERT* β shRNA inserts. Constructs were cloned into pLKO.1-Tomato vectors. A) Lentiviral construct sh control B) Lentiviral construct sh *hTERT* β 1 and 2.

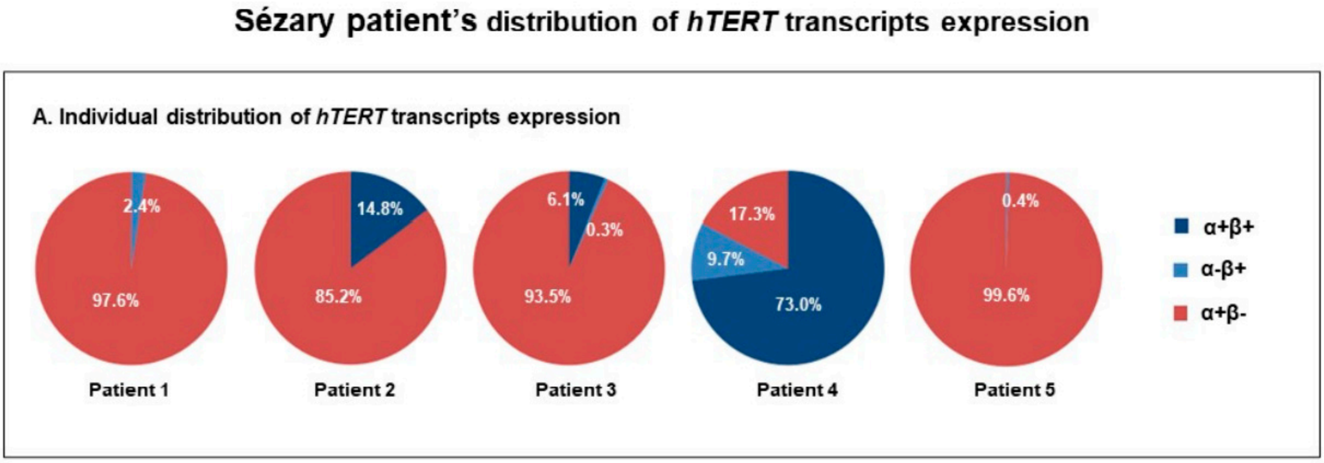

Supplemental Figure S3. Sézary patient's distribution of hTERT transcripts expression. *hTERT* RT domain transcriptome analysed in each five SS patients analysed. Distribution of *hTERT* transcripts expression. β+ variants are presented in red and β- variants in blue.

SS: Sézary Syndrome; hTERT: human telomerase reverse transcriptase; RT: reverse transcriptase.
